# A Multilingual Tool for Standardized Laboratory Biosafety and Biosecurity Assessment and Monitoring

**DOI:** 10.1089/hs.2022.0030

**Published:** 2022-12-08

**Authors:** Arnaud Orelle, Abdoulaye Nikiema, Arsen Zakaryan, Adilya A. Albetkova, Mah-Séré Keita, Mark A. Rayfield, Leonard F. Peruski, Antoine Pierson

**Affiliations:** Arnaud Orelle, MSc, was Biosafety and External Quality Assessment Coordinator, Integrated Quality Laboratory Services, Villeurbanne, France.; Abdoulaye Nikiema, PharmD, MSc, is a Senior Laboratory and Quality Management Systems Specialist, Integrated Quality Laboratory Services, Villeurbanne, France.; Arsen Zakaryan, DVM, PhD, is a Senior Laboratory Specialist, Integrated Quality Laboratory Services, Villeurbanne, France.; Antoine Pierson, PharmD, is Chief Scientific Officer, Integrated Quality Laboratory Services, Villeurbanne, France.; Arnaud Orelle is currently a Scientific Advisor, Scientific Direction, Lab'Science, Nazelles-Négron, France.; Abdoulaye Nikiema is also GHSA Program Manager, African Society for Laboratory Medicine, Addis Ababa, Ethiopia.; Mah-Séré Keita, MP, is Director of Programs, African Society for Laboratory Medicine, Addis Ababa, Ethiopia.; Adilya A. Albetkova, MD, PhD, Senior Laboratory Advisors, Division of Global Health Protection, Center for Global Health, US Centers for Disease Control and Prevention, Atlanta, GA.; Mark A. Rayfield, PhD, Senior Laboratory Advisors, Division of Global Health Protection, Center for Global Health, US Centers for Disease Control and Prevention, Atlanta, GA.; Leonard F. Peruski, PhD, is Chief, International Laboratory Operations, Division of Global Health Protection, Center for Global Health, US Centers for Disease Control and Prevention, Atlanta, GA.

**Keywords:** Laboratory safety, Biosafety protection, Risk management, Global Health Security Agenda, Biosecurity

## Abstract

Control of infectious diseases requires the handling of infectious materials by both clinical and public health laboratories with exposure risks for laboratory personnel and environment. A comprehensive tool for assessing the capacity to manage these risks could enable the development of action plans for mitigation. Under the framework of the Global Health Security Agenda action package for biosafety and biosecurity, the authors developed a tool dedicated to assessing laboratory biosafety and biosecurity. The Biosafety and Biosecurity Laboratory Assessment Tool (BSS LAT) assesses the status of all laboratory biosafety core requirements across 10 different modules. It consists of a standardized spreadsheet-based tool that provides automatic scoring. It is designed to support national, regional, and global efforts to strengthen biosafety in clinical, public health, and veterinary laboratories. The BSS LAT was first used in Burkina Faso in collaboration with the African Society for Laboratory Medicine and the US Centers for Disease Control and Prevention to support the country in strengthening their biorisk management system. Since then, it has been successfully used in other countries (ie, Armenia, Burundi, Cameroon, Ghana, Guinea, Kazakhstan, Liberia), various settings (medical and veterinary laboratories), and translated into several languages (eg, English, French, Russian). The BSS LAT is a multipurpose tool that assists with standardization of biosafety and biosecurity requirements for all laboratories working with infectious materials, serves as a self-assessment guide for laboratories to develop improvement plans and reinforce capacities, and serves as a training guide for individual laboratories and networks or at the national level. The BSS LAT can also be used as a monitoring tool for the assessment of biosafety and biosecurity across all laboratories working with infectious materials at the national, regional, and global levels.

## Introduction

The World Health Organization (WHO) International Health Regulations 2005 (IHR)^1^ is a legally binding international agreement designed to prevent the spread of disease. To support countries to fulfill IHR requirements, the Global Health Security Agenda (GHSA) was established in 2014, with a focus on the threats posed by infectious disease. The GHSA is a partnership of more than 70 countries and several international organizations, nongovernmental organizations, and private sector companies jointly working against infectious diseases.^2^ It leverages this multisectoral partnership to build and improve country capacity and leadership in the prevention and early detection of, and effective response to, infectious disease threats. The GHSA is composed of 11 action packages, including 1 on biosafety and biosecurity that is aimed at reducing the capability of dangerous pathogens to spread rapidly, within and across borders.^3^

Laboratory biosafety and biosecurity is of heightened interest due to concerns about accidental exposure to biological materials, deliberate misuse of biological materials, and emerging and reemerging biological risks.^4-6^ These risks continue to be magnified by globalization and by the continuous emergence or reemergence of zoonotic diseases. All infectious disease laboratories by the nature of their work deal with materials that, if inappropriately handled or improperly contained or stored, can pose serious concerns to health, including a public health event of international concern.^7^

Despite recent advancements in disease detection capacity globally, the design and implementation of associated laboratory safety and biosafety programs lags and is inconsistent due to a variety of factors including differences in national and local infrastructures, available funding and priorities, regulatory frameworks, and accessibility to expertise, training, and equipment resources.^8^

In line with the IHR and the GHSA initiative, worldwide laboratory capacities are expanding, which calls for concrete actions to improve laboratory biosafety and biosecurity practices to protect workers and the community. Biosafety and biosecurity have been elevated globally as a major concern, yet few action-oriented and practical tools for assessment and implementation exist. Different biorisk assessment instruments or guidelines to establish biosafety and biosecurity initiatives are available, such as the WHO *Laboratory Biosafety Manual*^9^; the Biorisk Assessment Model developed by Sandia National Laboratories^10^; the Danish biosecurity book, *An Efficient and Practical Approach to Biosecurity*^11^; the safety module of the Food and Agriculture Organization's Laboratory Mapping Tool^8^; the Biosecurity Self-Scan Toolkit;^12^ and the Biosecurity Vulnerability Scan.^13^

Interesting initiatives have also taken place but with limited scope. For example, the University of Indonesia developed a checklist tool for biorisk management,^14^ but it was only field tested in and adapted for Indonesia, thus limiting its applicability to other settings.

Although different instruments and guidance are available to assess biosafety and biosecurity programs within institutions or to identify biosecurity gaps at the national level, to our knowledge, there is currently no publicly available biosafety checklist for laboratory assessments. To this end, we developed a prototype assessment tool, based on Microsoft Excel, as part of the GHSA initiative to reinforce capacity and strengthen the laboratory system in Burkina Faso. In this article, we describe the tool, including how it is organized, its process of development and field testing, how to obtain it, as well as our experience using it and the impact it can have.

## The Biosafety and Biosecurity Laboratory Assessment Tool

The Biosafety and Biosecurity Laboratory Assessment Tool (BSS LAT) is based on a Microsoft Excel worksheet and consists of a standardized questionnaire. Using an Excel spreadsheet maintains an open and unlocked system, which can be modified; relies on a software system that is widely known and understood; is widely available commercially or has free counterparts that are widely available (eg, OpenOffice, LibreOffice, Google Docs); and facilitates the use and export of data into other formats, reports, or software.

### Organization and Modularity of the Tool

**Figure 1.**
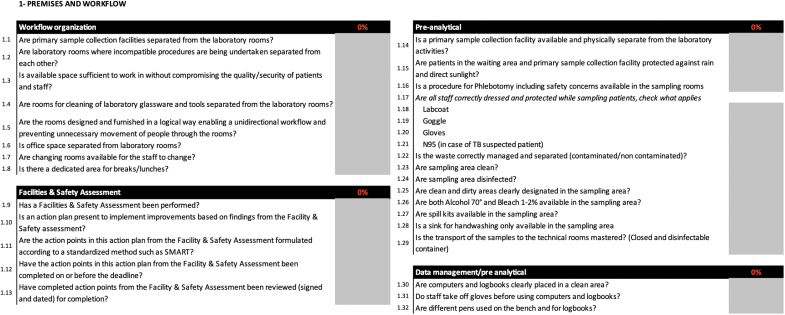
Example of questions listed in the tool. Closed answers and open comments are possible; the indicator score is calculated in real time as the user completes the questions.

The BSS LAT is divided into 8 nonnumbered and 10 numbered modules and has 538 questions, with an additional 108 questions for the biosafety level (BSL) 3-specific laboratory module. The tool begins with nonnumbered tabs, including a cover page, a list of abbreviations, and a general tab, which is used to gather basic information such as the name of the facility, information about assessors, contact details about the respondents, activities being performed in the laboratory as well as the associated workloads, GPS coordinates for geographic information system display of the results, and the laboratory BSL. Numbered topic-specific tabs follow, each including related indicators and questions (Figure 1).

The numbered BSS LAT modules were identified through the main biosafety and biosecurity topics commonly described in international references:^9,15-17^ (1) premises and workflow; (2) staff management and training; (3) good laboratory practices; (4) cleaning, disinfecting, sterilization, and waste management; (5) emergencies; (6) risk management; (7) documentation and regulations; (8) biosecurity; (9) other risks; and (10) BSL-3 laboratories. Each module contains various indicators and each indicator consists of several questions with closed answers (Table).

**Figure 2.**
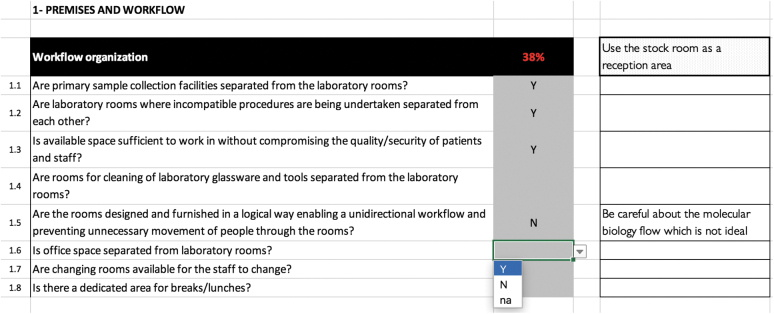
Example of “Premises and Workflow” module organization.

Each question and indicator can be completed either by using the closed drop-down menu or by filling in an open comment cell to provide more information about the specific question or indicator (Figure 2). The drop-down menu design facilitates the collection of quantitative information with closed questions (ie, only “yes” or “no” or “not applicable”). The tool provides automated direct score calculations for each of the following:

- Questions – for each question, the score is calculated as either 0% if the answer is unsatisfactory (almost exclusively when negative) or 100% if the answer is satisfactory (almost exclusively when positive)
- Indicators – for each indicator, the score is calculated as the average of all related question scores/component indicator
- Modules – for each module, the score is calculated as the average of all related indicator scores/component module
- Laboratory – an overall assessment score for the laboratory (also called the general indicator) is calculated as the average of all module scores

**Figure 3.**
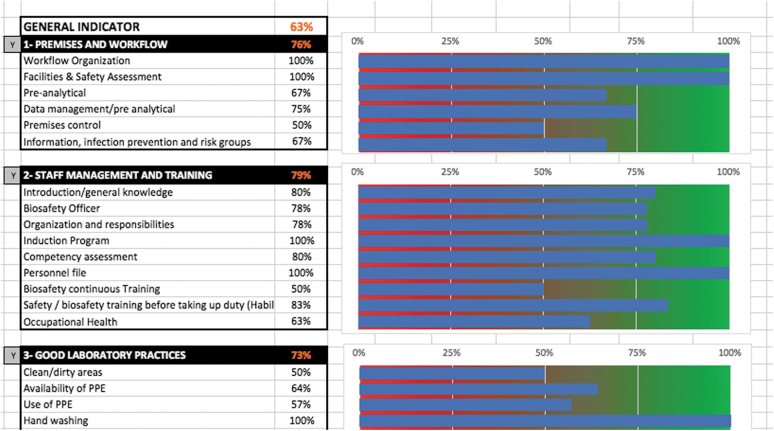
Overview of the summary page (and graphic depiction) after completing the tool.

Scoring is based on a percentage of a maximum score of 100%. The results of the checklist are presented in each module and in the summary tab using a color-coded system of red (0% to 50%), orange (50% to 80%), and green (80% to 100%). The tool includes summary pages, organized by indicator and module, with a graphic depiction and data export to a database or GIS mapping software or other software (Figure 3).

Although the sequential tabs ideally should be completed chronologically from left to right, it is not required. The cells to be completed are indicated with gray shading as a visual clue to show users where answers are needed. Users can save their session in the tool at any time and can resume later to facilitate the ease of the assessment and reporting process. It ends with a conclusion page where the assessor can enter findings and recommendations.

**Figure 4.**
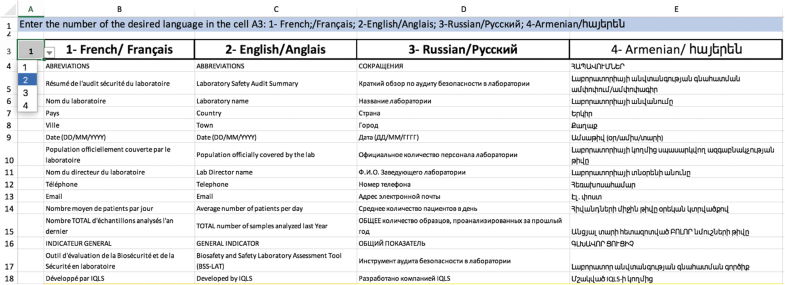
Overview of the language tab and language switch button (cell A3).

The BSS LAT is multilingual—currently available in English, French, Russian, and Armenian—and users can select the desired language from a drop-down menu. All language versions are contained in the same file, within the language tab (Figure 4). All versions are identical, with the exception of the Armenian version, which does not include a BSL-3-specific module, as no BSL-3 laboratories currently exist in Armenia.

### Experience Sharing and Recommendations of Use

The BSS LAT was developed to be as intuitive, user-friendly, and flexible as possible. The tool can be completed in 1 or more sessions (ie, the Microsoft Excel file can be saved at any time and continued later). All responses that lead to the calculation of an indicator score are entered using drop-down menus with a data validation mechanism to prevent mistakes. Although most of the questions in the tool are closed, each question has a comment field where assessors can add more details or specifics. The questions from the BSS LAT were formulated to limit subjectivity on the part of the assessor.

In addition, it is generally recommended that the assessor select a negative answer to questions if the element from the question is not fully in place, established, mastered, followed by all, or not fully completed (ie, for the question, “Is the following procedure available?,” the assessor should enter “no” if the laboratory did not finalize the procedure or put in into application yet; and for the question, “Is a personnel file available for all staff members?,” the assessor should enter “no” even if only 1 staff member (1 out of 10) does not have their personnel file available).

The recommended laboratory assessment process is as follows: (1) a courtesy visit to the laboratory management, (2) an explanation of the assessment process (stating that it is a participatory process, not punitive), (3) a detailed laboratory visit following sample flow (including sample reception, preanalytical phase, up to the waste management), and (4) completion of the tool during a plenary session. This can be most easily accomplished by presenting the tool (eg, in a meeting room using a video projector) to relevant, interested, and available staff.

The assessor should fill in all cells that have gray shading. Upon completion, it is recommended to present the immediate results to the assessed team and/or to the top management, and provide facilitated comments on the results. The entire assessment process for a laboratory is estimated to be a full work day. In our experience, however, the assessor must be flexible. No specific preparation is expected by the laboratory staff before the assessment. However, full availability is expected on the day of the evaluation from the managers and at least 1 person representing the technical staff. Depending on the size of the laboratory, the workload, the number of staff, the interest from assessed laboratory's staff, and the assessor's experience with the BSS LAT, the assessment process can be performed quicker or may take several days, especially if a plan of action is subsequently developed jointly by the assessment team and laboratory staff. As far as possible, we recommend approaching this exercise not as a simple assessment performed by an external party, but as an opportunity to sensitize staff to biosafety and biosecurity concepts, teach and train people on biosafety, provide supplementary documentation (eg, guidelines, standards, examples from other laboratories/settings), and answer questions on biosafety and biosecurity and in particular, in the current setting of the laboratory or the network.

The conclusion module/tab can be filled later by the assessor, along with recommendations enacted to the laboratory. The final BSS LAT, including conclusion and recommendations, should be provided to the assessed laboratory as soon as possible, for them to implement preventive and corrective actions. As much as possible, the assessor should organize recommendations in order by priority (eg, by associated risk, feasibility of the preventive or corrective action, budget required, and time required to establish the action).

## Discussion

### Origin and Development

Until recently, biosafety and biosecurity were not among the biggest concerns in laboratory science, especially in resource-limited countries. The BSS LAT was developed to gather and summarize the important aspects of biosafety and biosecurity in a laboratory setting, which have been unevenly treated depending on the source of the guidance or standard, as no official global standard currently exists.

The BSS LAT was built using the available international references and standards including the WHO *Laboratory Biosafety Manual* ^9^; the WHO *Biorisk Management: Laboratory Biosecurity Guidance*^16^; the World Organisation for Animal Health *Manual of Diagnostic Tests and Vaccines for Terrestrial Animals*^17^; the US Centers for Disease Control and Prevention (CDC) and the National Institutes of Health *Biosafety in Microbiological and Biomedical Laboratories*^15^; ISO 15190:2003, which pertains to medical laboratory safe practices^18^; the European Committee for Standardization (CEN) Workshop Agreement (CWA) 15793 laboratory biorisk management standard supplemented by CWA 16393^19^; the National Institutes of Health *Design Requirements Manual* for biomedical laboratories and animal research facilities^20^; the National Institutes of Health inspection checklist for BSL-3 laboratories^21^; and our knowledge and experience from the field and from various general assessments performed worldwide. At the time of the tool's development, the following guidance and standards were not in existence or published, but were considered during the adaptation of consecutive versions of the tool: ISO 45001, for occupational health and safety^22^; the ISO 35001, for laboratory biorisk management^23^; and the fourth edition of the WHO *Laboratory Biosafety Manual*.^24^ Several standards have since been updated, such as ISO 15190:2003, which has been replaced by ISO15190:2020.^18^

The BSS LAT was originally developed by Integrated Quality Laboratory Services in 2017 to perform assessments in Burkina Faso for the African Society for Laboratory Medicine as part of CDC-led efforts under the GHSA. The tool has since been field tested in an additional 7 countries, revised, and supplemented with additional questions and indicators. Before each use, the tool was refined through the removal of redundant elements, reformulation of questions (to increase precision and improve wording), correction of calculations, reorganization of some questions and indicators, development of new modules (ie, BSL-3 laboratory specific module), translation of the tool into several languages (ie, English, French, Russian, Armenian), and improvements to the layout.

Field testing occurred in Ghana in 2017 (which led to the addition of the English version of the tool and to field testing in a West African English-speaking country); in Armenia in 2018 (which led to the addition of the Armenian version of the tool and to dual testing in human and veterinary central and regional laboratories, in both clinical public and private laboratories); in Burundi, Cameroon, and Guinea in 2019 (Central and West French- and English-speaking countries, clinical public laboratories); in Kazakhstan in 2019 (which led to the addition of the Russian version of the tool, the development of BSL-3-specific module, and to field testing in an ex-Soviet country, at the central level); and in Liberia in 2019 (at central and regional levels, in a country recently affected by the Ebola virus disease). Importantly, all of the countries and laboratories were at different levels of initial biosafety implementation. Over the course of field testing, the BSS LAT benefited from being used by various experts and evaluators from different backgrounds and with different specializations, whose input we used to refine the tool by incorporating specific pathogens or scenarios into the formulation of questions. We are confident that this comprehensive field testing enabled us to deeply and extensively test the BSS LAT under broad and various conditions.

Through this iterative process, the BSS LAT has reached version 9.0 and is highly comprehensive, interactive, and robust. It provides a high level of confidence in its suitability and adaptability to a broad One Health spectrum of laboratories in different countries and continents.

The most recent version of the BSS LAT is available as a zipped file: https://www.iqls.net/docs/IQLS_Biosafety_LAT_v9_Oct2019_EN_FR_RU.zip. It consists of the open Excel tool, a printable PDF version, and a brief user guide.

### Impact

In our experience, all of the assessments we performed during the field testing period demonstrated that the tool can be used to introduce biosafety and biosecurity key concepts directly in the laboratory and among staff. Using this tool provides an opportunity to sensitize staff on biosafety and biosecurity issues and to answer practical questions. Results from the assessment provide direct onsite observations and recommendations that enable immediate preventive and corrective actions.

Additionally, our numerous BSS LAT tests demonstrated that the tool was effective in identifying the main gaps in a laboratory (or in a group of laboratories and/or at country level), which was a key factor in preparing a suitable plan of action to strengthen capacities and better target training needs.

If mastered at a high level, this tool could be applied on a regular basis to (1) monitor the biosafety and biosecurity level of laboratories across regions, a country, or countries; (2) design interventions; and (3) measure their impact. The detailed experience from Burkina Faso and how the BSS LAT was used in a global approach to strengthen biosafety and biosecurity capacities at national and regional levels is detailed in a joint publication.^25^ The assessment process was a decisive step in the selection of activities that were more likely to improve the laboratory system in Burkina Faso.

In the countries where we administered the BSS LAT a second time, after initial assessment and training, the tool provided a convenient measure of the impact of implemented activities and helped to quantify improvements in biosafety and biosecurity.

It is important to mention that although the BSS LAT has been found to be beneficial to laboratories and staff members, it remains a tool; it cannot replace the commitment by leadership to strengthen biosafety and biosecurity or to fund gaps and needs.

## Conclusion

The BSS LAT is simple to use and multilingual (including 3 United Nations languages all integrated into the same file). It relies on widely available software and is shared in an open format to facilitate adaptation (eg, specific to a country, specific standards, additional languages). It has been successfully tested in 8 countries to date, at the central and regional levels, in medical and veterinary laboratories, and in a specialized pathogen-specific laboratory setting. The BSS LAT has been used for various aims in different settings and at different levels, which (1) contributed to minor enhancements and simplifications to make it both comprehensive and straightforward and (2) clearly demonstrated important outcomes and benefits for strengthening biosafety and biosecurity capacities—the most detailed example of which is the experience from Burkina Faso.^25^ The BSS LAT was designed so that it can be used in any infectious disease laboratory at national or regional levels, and can be used by either external assessors or by laboratories for self-assessment.

Performing a biosafety and safety assessment using the BSS LAT has numerous benefits. It has a simple but effective checklist format and a standardized assessment process (especially helpful when conducting numerous assessments or assessing laboratories in different settings) that prevents oversights or bias from assessors. The BSS LAT facilitates a detailed and complete assessment that can be used to guide sustainable improvements as envisioned under IHR and the GHSA. It also provides laboratories with an objective assessment, as the results are automatically calculated and summarized in a table by topic, module, and/or graphic depictions. The tool can also be used for monitoring, either by the laboratory that was assessed (eg, auto-evaluation and follow-up) or by the assessor (ie, as individual, national, regional, or international body or partner). In addition to the practical assessment dimension of the tool, the BSS LAT can also be used to provide guidance and to sensitize laboratory staff to biosafety concepts, knowledge, and practices to strengthen biosafety awareness and practices that are increasingly critical globally.

In addition, the BSS LAT becomes a live monitoring tool after initial assessment to be used by laboratory management and staff to rapidly identify and prioritize what remains to be done and to document the implementation of corrective actions.

The BSS LAT can provide a strong foundation for countries or laboratory networks that would like to enhance or develop their biosafety capabilities or to develop national biosafety standards. For example, Kazakhstan used the BSS LAT to develop a national biosafety and safety checklist. In Burkina Faso, the tool was used to prioritize biosafety and biosecurity improvements across the national laboratory network. The BSS LAT is also in the process of being adapted for use in South America based on experiences from their COVID-19 response. These examples show the versatility of the tool and its adaptability in diverse settings.

The BSS LAT can be improved further and the authors welcome any suggestions. Any organization, national body, or bureau wishing to use the BSS LAT in a different language should contact the authors so they can support the translation process and include the new version in the tool's distribution package.
